# Supplementary figures and images for: Integrated insights into gut microbiota and metabolomic landscape in breast cancer patients undergoing adjuvant endocrine therapy
Source: mSystems. 2025 Aug 15;10(9):e00879-25. doi: 10.1128/msystems.00879-25 (PMC12455969; doi:10.1128/msystems.00879-25)

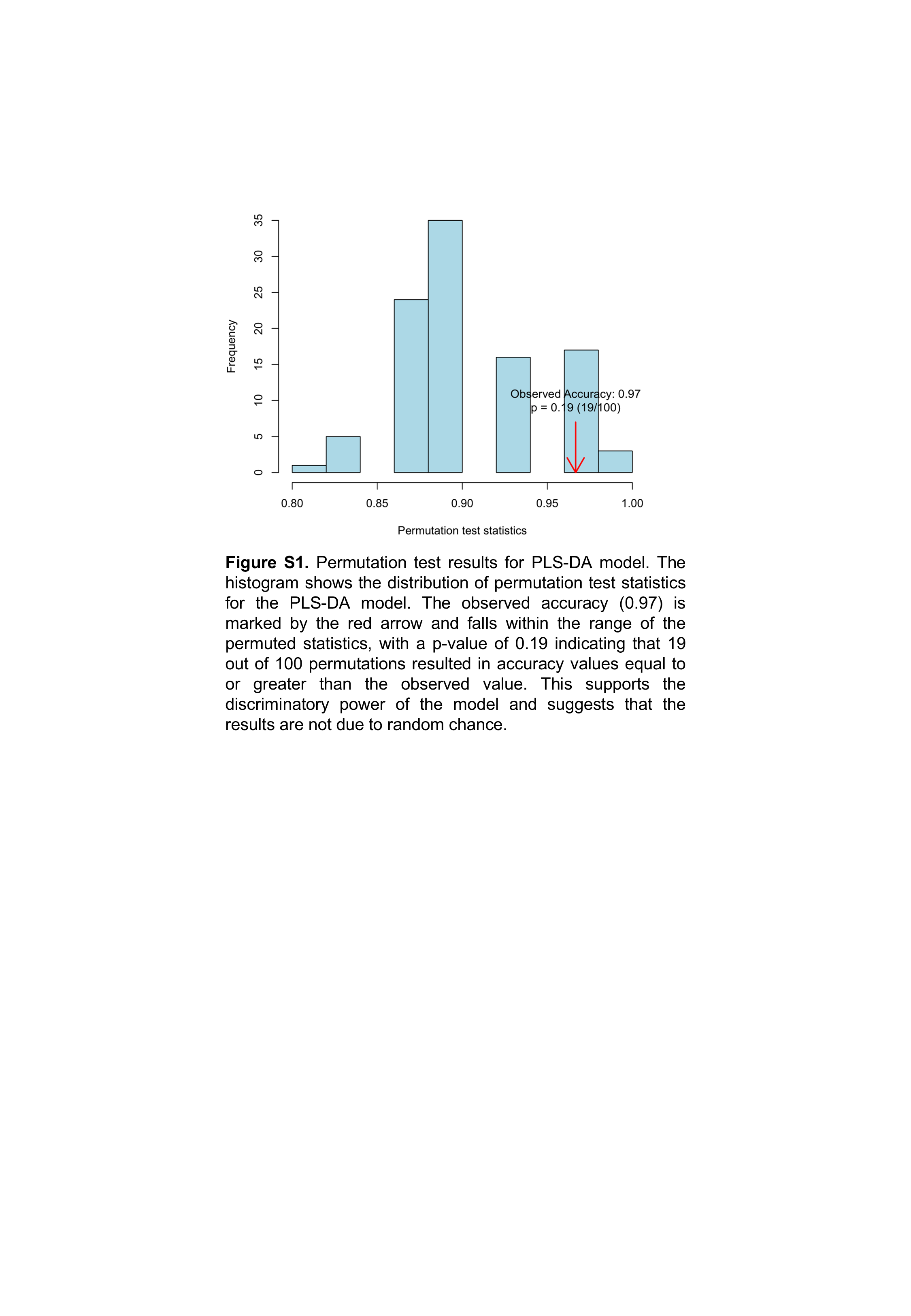

Supplement: Figure S1 — Permutation test results for PLS-DA model. [file msystems.00879-25-s0001.tif]

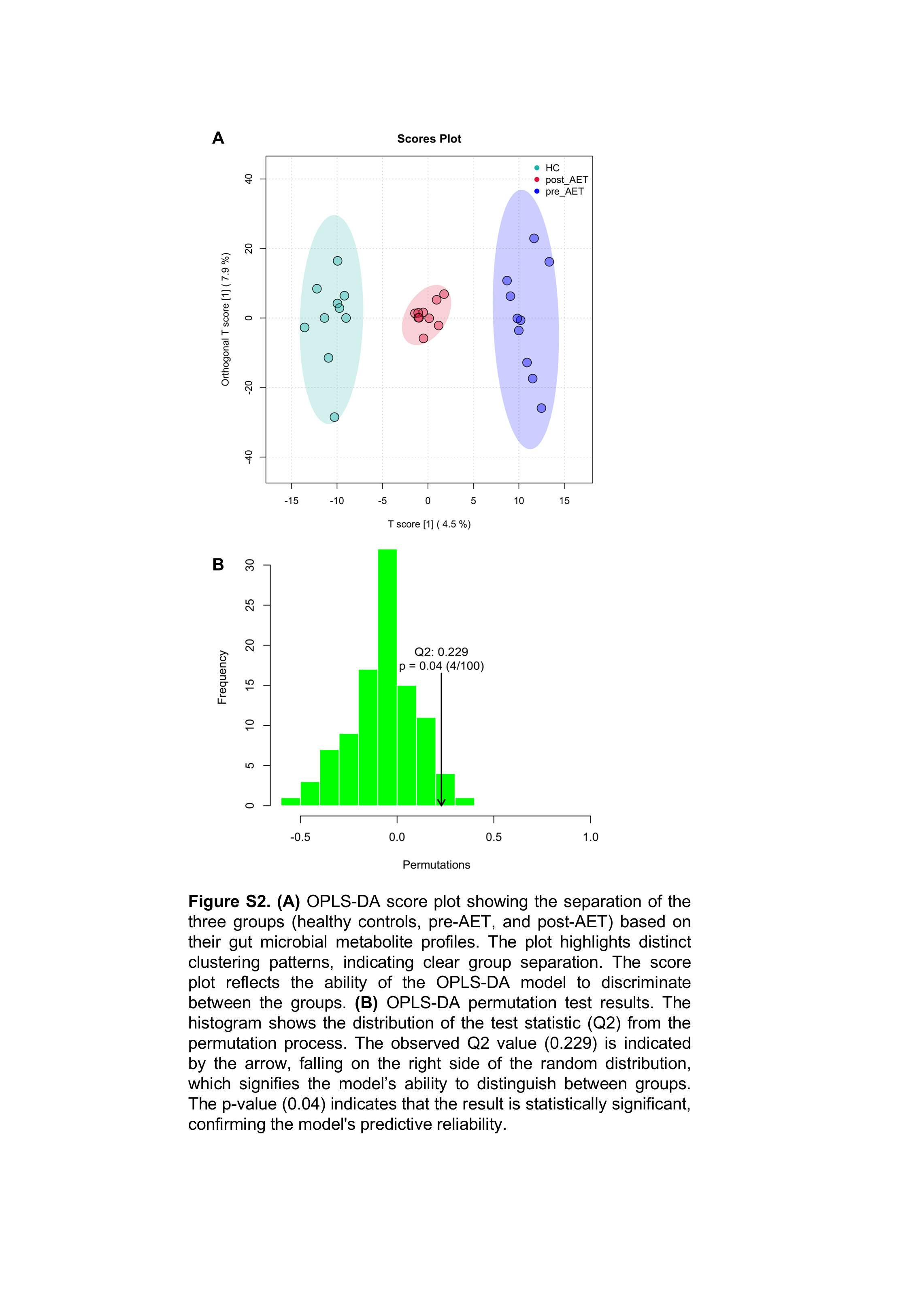

Supplement: Figure S2 — OPLS-DA score plot and permutation test results. [file msystems.00879-25-s0002.tif]
